# Effectiveness of topical caffeine in cataract prevention: Studies with galactose cataract

**Published:** 2010-12-08

**Authors:** Shambhu D. Varma, Svitlana Kovtun, Kavita Hegde

**Affiliations:** Department of Ophthalmology and Visual Sciences, University Of Maryland School of Medicine, Baltimore, MD

## Abstract

**Purpose:**

The primary objective of the study was to investigate the possible inhibition of cataract formation by topical administration of caffeine using the galactosemic rat model. It was hypothesized that caffeine will do so by acting as scavenger of reactive oxygen species known to be generated under hyperglycemic conditions.

**Methods:**

Cataract was induced by feeding young rats a diet containing 24% galactose for a period of 25 days. A control group of such rats was treated with a placebo eye drop preparation containing hydroxy propyl methyl cellulose as a wetting agent. In the experimental group, the rats were treated with the above preparation mixed with 72 mM caffeine.

**Results:**

Administration of caffeine eye drops was found to significantly inhibit the onset as well as the progress of cataract formation. By day 25 on the galactose diet, all the animals in the control group developed advanced white opacity spread over the entire area of the lens. In the caffeine group, the formation of such opacity remained strikingly inhibited. The lenses remained largely transparent. The transparency data paralleled the higher concentration of glutathione maintained by caffeine treatment. Its levels in the placebo group were 0.8, 0.5, and 0.4 µmoles/g lens wt. on days 5, 15, and 25 against a consistent basal control value of ~3 µmoles/g over the entire period. In the caffeine group, the corresponding values were nearly 3 µmoles/g till day 15, but decreasing to ~2 µmoles/g on day 25. The levels were hence significantly higher than in the caffeine untreated group, remaining relatively closer to the basal controls. In addition, the compound was found effective in inhibiting morphological changes induced by galactose.

**Conclusions:**

Micromolar amounts of topical caffeine have been found to be significantly effective in inhibiting the formation of galactose cataract, strongly suggesting its possible usefulness against diabetic cataracts. The effects are attributed to its ability to prevent oxidative stress and consequent maintenance of tissue metabolic and transport functions, in addition to preventing the induction of apoptosis.

## Introduction

Caffeine is one of the most common nutraceuticals derived from many common beverages such as coffee, tea and various colas. In addition, it is a common food additive such as that in chocolates, cakes, and candies and some other food products.

Conservatively, the average human consumption of this alkaloid derived from coffee drinking alone ranges from approximately 250–400 mg per day, the concentration in an average cup of coffee being about 200 mg/12 ounce [1]. Additional significant amounts are derived from soft drinks and tea. The US Food and Drug Administration (FDA) lists this as a generally safe compound for human consumption, the LD_50_ being substantially high (10 to 15 g of a single dose) [2], equivalent to 80–100 cups of coffee. Achieving lethality is difficult. Additionally, regular use of this compound, mostly derived from coffee drinking, has been reported to provide several very significant health benefits such as the reduced incidence of of Alzheimer and Parkinson disease [3-7], senile dementia, and prevention of neural degeneration in general and associated loss of cognitive performance [8-10]. Recent reports also suggest that it decreases the risk of the development of Type 2 diabetes [11,12] loss of liver function and cirrhosis [13,14] and certain cancers [15-20]. Previous reports suggesting an association between regular use of caffeine and development of hypertension has also been significantly toned down in view of recent epidemiological findings. The association between hypertension and coffee intake could not be established in a study based on at least 155,000 nurses [21]. A Harvard study with 128,000 people concluded that coffee consumption is also not associated with coronary heart disease unless it is used in combination with cigarette smoking and excessive use of alcohol [22]. These and many other such positive reports point out to the desirability of further studies on the mechanism of action of this ubiquitously prevalent nutraceutical. Interestingly, studies on its possible beneficial and therapeutic effects against the aging eye diseases have been very limited except some recent preliminary reports from this laboratory suggesting that it has a potential to slow down cataract progression. The suggestion has been based on the in vitro organ culture experiments showing its effectiveness in offering protection against lens damage caused by UV exposure, as reflected by the severe inhibition of the active cation transport process as well as the depletion of GSH. Incorporation of caffeine in the culture medium protects against the induction of these deleterious effects [23,24]. It also prevents the loss of tissue transparency associated with UV exposure. Such a loss of transparency in vivo would directly interfere with transmission and refraction of light coming into the eye and its focusing on the retina. Interestingly, in a preliminary study, caffeine has also been shown to prevent phototoxic damage to retina [25]. The anti-cataractogenic potential of the compound in vivo is suggestible also by preliminary studies showing that formation of cataracts in rats maintained on a high galactose (24%) diet is significantly inhibited if the galactose diet is fortified with 1% caffeine [26]. However, to rule out the possibility that such an effect of caffeine could have been systemic in nature, it was considered necessary to investigate if caffeine could be effective when administered topically in the form of eye drops. Also, such topical application of the compounds is well known to enhance their local pharmacological effects in the eye, in addition to avoiding any unwanted systemic effects.

## Methods

Most of the routine chemicals used were procured from Sigma Aldrich Chemical Company, St. Louis, MO. N-methyl-14C-caffeine was obtained from Perkin Elmer, Boston, Massachusetts (Catalog # NEC4120; Perkin Elmer). DAPI (4',6’-diamidine-2-phenylindole) was obtained from Roche Diagnostics, Indianapolis, IN. Fluorescein-12-dUTP (Fluorescein-5(6)-carboxaminocaproyl-[5-(3-aminoallyl)-2´-deoxy-uridine-5′-triphosphate) was obtained from Life Technology, Carlsbad, CA. Sprague Dawley rats were obtained from Harlan Laboratories, Indianapolis, IN.

### Determination of caffeine penetration in the aqueous humor and lens

An eye drop preparation was made by adding hydroxypropyl methyl cellulose (0.9%, 3,000 centipoise) to 72 mM aqueous solution of caffeine water. The placebo was made by substituting caffeine with 72 mM NaCl. In penetration studies, ^14^C_1_-caffeine was added to above caffeine solution acting as a tracer. This preparation (20 µl) was then instilled into the cul-de-sac of six rats kept anesthetized with ketamine (80 mg/kg) and xylazine (10 mg/kg). Aqueous humor samples were withdrawn from both the eyes at time points of 30, 60, 120, and 180 min using an insulin syringe. The aqueous samples withdrawn were transferred to a conical microfuge tube and lightly centrifuged to collect the fluid at the bottom. Measured volumes (5 µl) of such samples were then transferred to a liquid scintillation cocktail (Econo-safe^TM^; Research Products International Corporation, Mount Prospect, IL) and their radio activities determined. The concentration of the caffeine in the aqueous was then determined by reference to the specific activity of ^14^C in the eye drop preparation which was kept near 250 cpm/nanomole. The lenses were isolated after taking out the aqueous humor, rinsed with saline, weighed and then homogenized in 75 µl of water. The homogenates were then quantitatively transferred to the scintillation cocktail vial and radioactivity determined as above. The amount of caffeine present in the lenses was then determined with reference to its specific activity in the eye drop preparation.

### Assessment of cataract inhibition

The initial objective of the study was to ascertain the effectiveness of caffeine eye drops against development of early cataracts. This was done by maintaining rats weighing about 50 g on a 24% galactose diet for 5 days and administering the eye drops containing caffeine five times a day at 90 min intervals till four days. On the fifth day, drops were given only twice, 2 h before euthanization. In the control group maintained on the galactose diet, the eyes were treated with the caffeine free placebo drops. The eyes were examined ophthalmoscopically for appearance of cataracts. Peripheral cortical cataracts became visible in all the placebo treated animals by day 4. On day 5, the animals were euthanized by CO_2_ inhalation, eyes were then enucleated and lenses dissected out by posterior approach and transferred to saline maintained at 37 °C till photographic documentation of cataract. The time between isolation of the lens and photography was less then 15 min. The photographs were taken after placing the lenses on a Millipore metallic grid using transillumination.

Subsequent experiments were then conducted to ascertain the possibility of delaying the formation of cataracts even further by such topical application. Hence in these experiments, treatment with the placebo or the caffeine eye drops was continued for either 15 or 25 days. The number of animals in each group was at least six. Lenses were then isolated as described above. One lens isolated from each of the animals was quickly frozen for later determination of glutathione (GSH). The contralaterals were used for photography as described above. A semi quantitative assessment of the densitiy of opacity as present on day 25 was also done by Adobe photoshop (Adobe Systems Inc., San Jose, CA) using program number CS3.

Random samples of lenses after photography on day 15 were fixed in PBS buffered 10% formalin and subsequently paraffin embedded for sectioning. The microtome sections were then stained with H&E for examining the general structure of the tissue. The distribution pattern of the nucleated cells was monitored by staining with DAPI. The presence of apoptotic cells was detected by terminal deoxynucleotidyl transferase dUTP nick end labeling (TUNEL) staining based on terminal deoxynucleotidyl transferase catalyzed labeling of the 3′-OH of the damaged DNA with fluorescein-12-dUTP.

## Results

**Figure 1:**
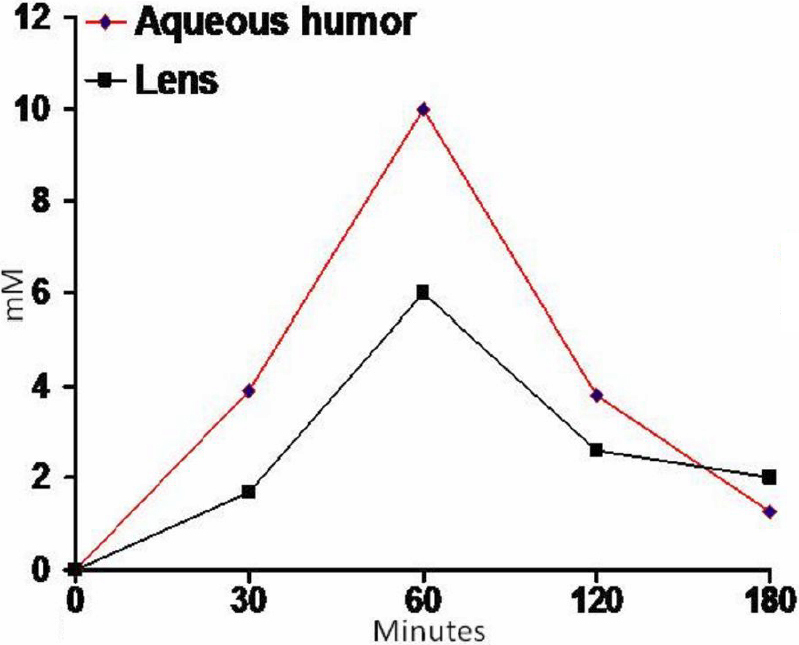
Intraocular penetration of Caffeine in aqueous and lens. Twenty microliters of the caffeine eye drop preparation labeled with 14C_1_-caffeine was instilled in the eyes of anesthetized rats. Samples of aqueous were prepared as described in the text and their radioactivity determined by liquid scintillation counting. The concentrations of the caffeine were then calculated with reference to the specific activity of caffeine in the eye drop. The results are described as Means of four aqueous and four lens samples. The standard deviation at each point was less than 0.35 mM.

Since the objective of these experiments was to determine if caffeine would be effective in inhibiting cataract formation when applied topically, initial experiments were designed to study the extent of its penetration through the cornea into the aqueous and the lens after its topical application in the form of an eye drop. Such information was not available in the literature. The amount of caffeine contained in the drop applied (20 µl) was 279 µg. In case of its theoretical 100% availability and its distribution in the aqueous humor with a normal volume of ~10 µl, the resulting aqueous concentration would be ~144 mM. However, as expected, this was not the case due to the inherent unavailability of the entire amount instilled because of the animals closing their eyes after the application of the drops at room temperature (even though under mild anesthesia) and consequent expulsion and leakage of significant portions of the applied drops through the palpebral fissure and the nasolacrimal duct. However, the application was found adequate enough to raise caffeine concentration significantly in the aqueous as well as in the lens. As summarized in Figure 1, the aqueous levels were 3.9,10, 3.8, and 1.8 mM at 30, 60, 90, and 120 min time points. The corresponding levels in the lens were 1.8, 6, 2.4, and 2 mM. Based on the previous in vitro studies showing inhibition of the ROS induced damage to the lens by caffeine [23,24], these levels were considered adequate for judging its effectiveness in inhibiting the cataract formation. That was indeed found to be true.

**Figure 2:**
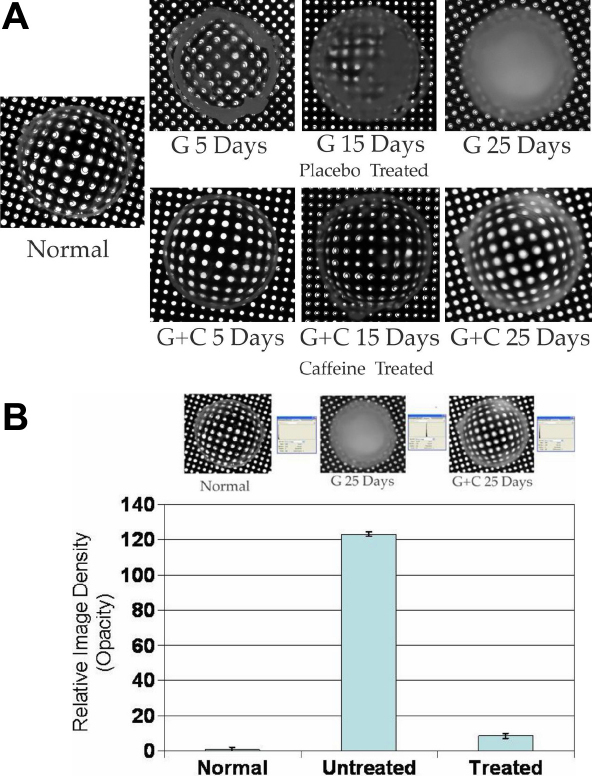
Cataract development in galactose fed rats. Preventive effect of caffeine. At various stages of cataract development the animals were euthanized by CO_2_ inhalation, lenses dissected out and placed on a Millipore metallic grid with uniform holes. Photographs were then taken in dark with retro-illumination. **A**: Cataract is indicated by the obstruction in light transmission through the grid holes because of the noticed opacity. **B**: Semiquantitaive evaluaton of the density of opacity (**G**: galactose diet; **C**: caffeine treated).

As shown in Figure 2A, application of such a drop (no radioactivity being present in these eye drops) five times a day to the animals maintained on 24% galactose diet had a significant inhibitory effect on the onset as well as the progression of cataract formation. A well discernible equatorial cortical cataract represented by the white peripheral ring formed in 4 to 5 days in the rats given the galactose diet and treated only with the placebo eye drops. On the contrary, in the group where animals were treated with the caffeine eye drops, development of such opacity was nearly completely abolished, the lenses remaining essentially clear. The anti-cataractogenic effect of caffeine was hence more specifically demonstrated here than that in the earlier preliminary experiments [26] where the galactose diet contained 1% caffeine. The present experiments therefore effectively rule out the possibility that the protective effective of caffeine observed earlier could have been a systemic effect. In addition, the effectiveness of the caffeine noticed by topical administration of only micro molar amounts of caffeine points out further its pharmacological suitability and rules out the possibility of any toxicity when used topically.

Hence the above investigations were extended further to examine if the anticataractogenic effect, as observed in the early stages of galactose feeding, can remain sustained, delaying the formation of cataracts even further. Hence in these experiments treatment with the eye drops were extended up to 25 days, the time required for complete maturation of the galactose cataracts in the control (caffeine-free) group. As shown in Figure 2A, the degree of opacity by day 15 in the galactosemic animals given the placebo eye drop, as expected, was much more advanced, well marked and much more extensive than that noted in the short-term experiments where the experiments were terminated on day 5. The severity of opacity in the placebo group advanced further by day 25, the opacity covering the entire lens. On the contrary, in the case of animals treated with the caffeine eye drops, the lenses were still transparent. The effectiveness of treatment was further analyzed (Figure 2B) semi quantitatively using Adobe Photoshop (CS3); showing that the density of opacity in the lens of the placebo group was about 15 times higher in the caffeine group. The lenses in the caffeine treated group were still largely clear except some peripheral opacity not adequate to hinder any significant light transmission.

**Figure 3:**
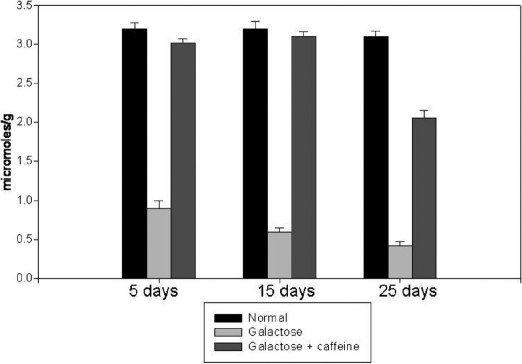
Levels of glutathione in the lenses of different groups. The values are expressed as Means±standard deviation. The depression of GSH in the galactose alone group as compared to the normal as well as when compared to the galactose plus caffeine group is highly significant (p values ≤0.001) at all points of observation. The values in the galactose plus caffeine group were also significantly higher than the galactose alone group. By 25 day the GSH value in the galactose plus caffeine group was lower than in the control groups, but was still higher than in the galactose alone group and adequate to maintain lens transparency barring the periphery.

The preventive effect against opacification was also apparent in terms of the levels of GSH summarized in Figure 3. In the five day experiments, the level of GSH in the galactose fed animals treated with the placebo decreased to about 24% of the basal controls. In the caffeine treated group it was more than three times of the placebo group, remaining still close to 90% of the basal controls. On day 15 also the levels in the caffeine group remained close to the basal controls. In the caffeine untreated group the levels remained depressed to about 15% of the basal controls. On day 25, the level of GSH was still maintained at a level of 65% of the basal control, the level in the untreated group being only 12% of the basal controls. The GSH level in the caffeine treated groups remained much closer to the basal controls than in the untreated groups where it underwent a substantial decrease.

**Figure 4:**
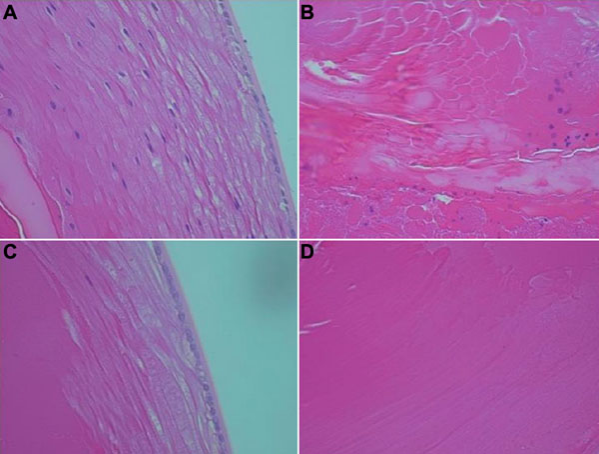
H&E profile of the lens sections. The upper panel (**A** and **B**) represents lenses in rats given the galactose diet treated only with the placebo. Apparent is the retention of nucleus in the fiber cells due to defective cellular transition. The nuclei are also clearly pyknotic. The central area of the tissue (**B**) also contains some nucleated cells. Other cells are swollen and detached form each other. These abnormalities were inhibited in the group treated with caffeine eye drops as shown in the lower panel (**C** and **D**).

The effect of caffeine was also apparent by H&E histology as presented in Figure 4. In the galactosemic group not treated with caffeine, the subepithelial region including the deeper cortical region consisted of numerous nucleated fibers indicative of defective differentiation of the epithelial cells to fiber cells, the deeper nuclear layer containing much swollen denucleated cells. The overall structure of this region is non-homogenous represented by denser opacity as compared to the cortex, as also apparent in Figure 2. As can be seen in Figure 4C,D, lenses of the galactosemic animals treated with 1.4% caffeine eye drops (ED) maintained a normal cellular structure consisting of a single layer of epithelial cells followed by well aligned non-nucleated fiber cells. The central portion of the tissue consisting of compacted fibers also remained homogenous.

**Figure 5:**
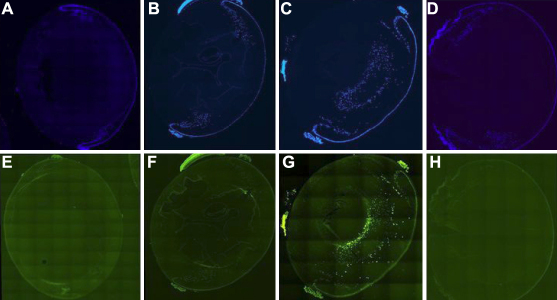
Induction of apoptosis. Prevention by caffeine. The pictures represent the sections of the lens stained with DAPI (**A**-**D**) and F-UTP (**E**-**H**) Sections **A** and **E** represent normal lenses. Sections **B** and **F** represent the lenses from normal rats treated with caffeine eye drops. Sections **C** and **G** represent lenses from galactose group treated with placebo. Sections **D** and **H** represent caffeine treated group. Photographs **C** and **G** indicate defective transformation of the epithelial cells into fiber cells, indicated by retention of nuclei and/or apoptosis. Sections **D** and **H** prove that caffeine inhibits such apoptotic process.

Figure 5 represents the sections stained with DAPI (Figure 5A-D) and F-dUTP (Figure 5E-H). As apparent by the DAPI staining of the normal lens section (Figure 5A) most of the nucleated cells are present in the bow zone of the tissue. Treatment with caffeine eye drops has no effect (Figure 5B), the cellular configuration remaining similar to the blank controls. On the other hand, in the case of galactose fed animals the distribution of the DAPI cells was highly abnormal (Figure 5C) with high abundance of the nucleated cells in the deeper cortex. That these cells indeed are apoptotic was proven by TUNEL staining using F-dUTP (Figure 5G). In the lens from galactosemic animals treated with the caffeine eye drops (Figure 5H), the distribution of the DAPI positive cells was similar to that in the normal non-galactosemic controls, showing the effectiveness of caffeine in inhibition of abnormal cell maturation induced by galactose. Apoptotic cells could also not be detected in the normal controls without and with caffeine (Figure 5E,F). As shown in Figure 5H, apoptosis was completely inhibited by the caffeine regimen of the galactosemic animals. It should be pointed out that caffeine has been reported to induce apoptosis in certain cancer cells on exposure to high intensity ionizing radiation, a process helpful in getting rid of the cancerous cells. However, caffeine does not seem to induce any apoptosis in the lens cells as has been found in studies with other noncancerous cells including that in the cornea and indeed its effectiveness in inhibiting the apoptosis in the lens indicates that it is able to inhibit DNA damage caused by oxidative stress, similar to the effect of pyruvate reported previously.

## Discussion

Since oxidative stress induced by reactive oxygen species plays an important role in the genesis of cataracts, it is highly likely that its formation can be attenuated or even prevented by use of certain oxy-radical scavengers. This is strongly apparent by several in vitro as well as in vivo studies with animal models. For example, the formation of cataracts in rat pups induced by oxidative stress triggered by administration of sodium selenite as an oxidant has been found to be inhibited by subcutaneous/intraperitoneal injection of ascorbate, pyruvate and alpha ketoglutarate [27-29].We have recently reported that caffeine is also highly effective in preventing selenite cataract formation. Formation of cataracts in the diabetic and galactosemic rats is also prevented by oral administration of flavonoids and other aldose reductase inhibitors, acting as antioxidants [30,31]. This has also been found in diabetic mice where cataract development is prevented by pyruvate as an antioxidant and metabolic stimulant, independent of its action as aldose reducatase inhibitor [32-34]. The pharmacological use of most of these compounds for treating and preventing human cataracts however still remains to be realized. This is because of their much higher dilution in the human body when administered orally with the consequence of only low concentrations available in the ocular tissues. In addition they are effectively metabolized and used by other body tissues before reaching the eye in adequate concentration. A larger dose may even have undesirable systemic effects. While they can be administered topically, their instability in topical eye drop preparations is also a significant limiting factor. This is due to their susceptibility to auto-oxidative degradation. In addition their auto-oxidation can generate products which can act as pro-oxidants and protein cross linkers. It is therefore considered desirable to examine the feasibility of preventing cataract formation by oxyradical scavengers that are more stable, even when incorporated in eye drop preparations and their reaction products generated by reacting with ROS are also not toxic. With this in view, we are studying the possibility of inhibiting cataract formation by use of caffeine. The compound is easily available and is much more resistant to auto-oxidation. Its reaction with ROS is accompanied with the formation of its 8-hydroxy derivative [35-37], with structure similar to urate, another ROS scavenger. In addition, it is known to be well tolerated by humans even when consumed in significant amounts as a common nutraceutical used over the entire life span. Interestingly, its consumptions has been shown to provide several significant beneficial health effects mentioned earlier, including its favorable effects against nerve damage associated with highly disabling Alzheimer and Parkinson disease. Oxidative stress is known to be a causal factor in the etiology of both these diseases; with the difference that in the case of lens such oxidative stress is more pronounced. This is due to the continued light penetration into the eye during the long periods of photopic vision and the consequent prolonged exposure of the lens to oxy-radical species generated continuously by pseudo catalytic photochemical reactions [38,39]. We have recently reported that such damage to the lens in culture induced by UV irradiation is significantly preventable by caffeine. That the preventive effect is due its oxy-radical scavenging property has been affirmed by ESR studies as well as by parallel studies with other well established ROS Scavengers such as Tempol [24]. Additionally, it has been found to protect lens cultured in dark with ROS generated by incorporation of soluble iron into the medium [40]. Further studies are therefore considered desirable to further ascertain its anti-cataractogenic effect in vivo.

Encouraged with the earlier preliminary findings [26] suggesting that caffeine could inhibit cataract formation induced by high levels of sugars, the present investigations were undertaken to examine its possibility further by administering it directly in an eye drop. This was hypothesized to be more effective even at doses lower than the oral dose used earlier, with the eventual objective of further pharmacological evaluation. It is also hypothesized to overcome the concern of any undesirable systemic effect possible if used orally in relatively larger amounts. Indeed its presence in coffee has been controversially suggested to transiently elevate the intraocular pressure by 1 to 2 mm in patients with existing glaucoma [41-43]. However the results are considered insignificant [42,43]. A deviation of 1 to 2 mM can take place even after water drinking [44,45]. Indeed, the potential of caffeine to induce glaucoma has now been well disproven by a very high powered prospective cohort study with 79,120 women and 42,052 men [46]. A suggestive increase of 1–2 mm in the intra-ocular pressure has also been discounted because of a normal variability of a few millimeters in the measuring technique itself. It should also be mentioned that coffee and caffeine are not equivalent. A well recognized scientific study with 400 mg of pure caffeine administered orally has also been found entirely ineffective in either raising the intra-ocular pressure or inhibiting aqueous flow measured by fluorophotometry [47]. Hence the present findings on the inhibition of cataract formation by caffeine are considered to provide adequate base line information for further studies with view points of pharmacology and toxicology. The importance of scavenging ROS and maintaining GSH levels has been previously suggested also by several previous papers [48,49]. Examination of the effectiveness of caffeine against cataract formation was done by topical application of 279 μg of caffeine administered 5 times a day. Hence the amount administered each time was much less than even the LD50 of 200 mg/kg to rats required to be administered in a single dose. The starting weight of the rats was 50 g. Hence, the total dose given per day was only 1.4 mg. This was also much lower than LD50. Only a small fraction of this actually got into the aqueous humor. As shown graphically in Figure 1, despite application of caffeine in such small amounts, cataract formation was dramatically inhibited. In the animals treated with the placebo eye drops, the early cataract in the form of equatorial and cortical opacity was notable by 4th to 5th day after putting the animals on the galactose diet, similar to that reported previously. This was significantly absent in the group given the caffeine eye drops. As expected, by day 15 the cataract became much advanced in placebo group, the opacity in these lenses being more severe and covering a much larger area of the lens, as compared to that on day 4. In addition to the presence of clearly white opacity, covering at least 50% of the lens surface or more, other areas also became hazy as apparent photographically. But in the group treated with the caffeine eye drop, lenses were still largely transparent without any significant haze or opacity. The protective effect remained sustained by continued treatment at least till 25 days when the experiments were terminated.

Caffeine has previously been reported to induce cellular apoptosis in some experimental conditions such as on incubation of certain transformed cancerous cells. It was therefore considered important to examine the possible presence of such apoptotic cells in the lenses of the placebo and caffeine treated groups. As expected no apoptotic cells were detected in the animals on galactose free diet. In the galactose fed group however, significant number of cells were TUNEL positive. However, in the caffeine treated group, apoptotic cells were not detected, as previously shown in cornea [50]. The results therefore clearly demonstrate that caffeine does not induce apoptosis in lenses. In addition, it also stops the apoptotic process associated normally with cataract formation including that in diabetes [34]. Since oxy-radical formation is well known to induce DNA damage, it is highly likely that the TUNEL positive cells in the galactosemic lenses represent DNA damage caused by its oxidative stress. The preventive effect of caffeine is therefore apparently related to its ability to detoxify reactive oxygen generated under hyperglycemic condition. The significance of other modes of its action such as its effect of maintaining higher levels of c-AMP by inhibiting phosphodiesterase in the lens remains to be verified at present. However, the findings that caffeine prevents cataract formation induced by a strongly potent cataractogenic agent such as galactose in vivo and against UV induced ROS damaged noted earlier suggest the suitability of its use pharmacologically, particularly as an eye drop for cataract prevention or delay. The agent as used is hypothesized not to be toxic also in view of the well known human tolerance to this and other similar alkaloids. Further mechanistic and pharmacokinetic studies are hence in progress, including studies with the diabetic models.
